# Aneurysmal bone cysts of the spine

**DOI:** 10.1007/s00586-012-2510-x

**Published:** 2012-10-01

**Authors:** Mehmet Zileli, Hasan Serdar Isik, Fatih Ersay Ogut, Merih Is, Sedat Cagli, Cem Calli

**Affiliations:** 1 Department of Neurosurgery, Ege University, Izmir, Turkey; 2 Department of Neurosurgery, Ordu University, Ordu, Turkey; 3 Department of Neurosurgery, Gaziosmanpaşa University, Tokat, Turkey; 4 Depertment of Neurosurgery, Lutfi Kirdar Kartal Training and Research Hospital, Istanbul, Turkey; 5 Department of Radiology, Ege University, Izmir, Turkey; 6 1421 sok 61/5, Alsancak, Izmir, 35230 Turkey

**Keywords:** Aneurysmal bone cyst, Spine tumor, Spinal fusion, Tumor recurrence

## Abstract

**Purpose:**

Aneurysmal bone cyst is a benign, relatively uncommon lesion, representing 1.4 % of primary bone tumors. The vertebral column is involved in 3–30 % of cases. This report describes clinical characteristics and treatment results of 18 patients with aneurysmal bone cyst of the spine.

**Methods:**

Between 1991 and 2008, 18 patients with aneurysmal bone cyst of the spine were surgically treated in our department. The clinical records, radiographs, histologic sections, and operative reports were analyzed.

**Results:**

There were 11 male and 7 female patients; mean age was 22.1 years (range 7–46 years). Localizations were cervical (3), cervicothoracic (2), thoracic (3), lumbar (4), and sacrum (6). Tumor was localized on the left side in 11 cases, on the right side in 2 and at midline in 5 patients. The two most common clinical features were axial pain (14 patients) and radicular pain (8 patients). Neurological signs were paraparesis in 3, monoparesis in 6. Mean duration of symptoms was 9 months (range 3 months–3 years). All patients underwent surgery: total removal was performed in 13 patients and subtotal resection in 5. Posterior (11), anterolateral (1), or combined anterior-posterior (6) approaches were used. Mean follow-up duration was 112.3 months (range 4–21 years). We detected four recurrences in subtotal excision group (4/5), and one recurrence in total excision group (1/13).

**Conclusion:**

Treatment options for aneurysmal bone cysts are simple curettage with or without bone grafting, complete excision, embolization, radiation therapy, or a combination of these modalities. Radical surgical excision should be the goal of surgery to decrease the recurrence rate. Recurrence rate is significantly lower in case of total excision.

## Introduction

Aneurysmal bone cyst (ABC) is a benign, tumor-like, highly vascular, locally aggresive, and relatively rare osteolytic lesion of unknown etiology [1]. The lesions primarily occur in the first two decades of life, with slight women predominance [2, 3]. After osteoid osteoma and osteoblastoma, ABC is the third most frequent benign bone tumor. Primary ABCs represent 1.4 % of primary bone tumors and the vertebral column, especially lumbar area and posterior elements are involved in 3–30 % of cases [4, 5]. Pain is the most common complaint, occurs especially at night, and it is localized to the site of the lesion. Direct radiographs, computed tomography (CT), and magnetic resonance imaging (MRI) help in diagnosis. Direct radiographs show an expansile osteolytic cavity. Fluid–fluid levels may be seen on both CT and MRI [6]. Management of ABCs of the spine is controversial. Options of treatments of ABCs in spine are surgical resection, radiation therapy, cryotherapy, and embolization [5, 7].

The purpose of this study is to describe the incidence, clinical presentation, diagnostic and therapeutic options, recurrence rate of the patients with ABC of the spine in our institute.

## Methods

Eigtheen patients with ABCs in the spine were surgically treated in our department between 1995 and 2010. The clinical records, radiographs, histologic sections, and operative reports were analyzed. The mean follow-up duration was 112.3 months (ranged from 4 to 15 years).

## Results

There were 11 male and 7 female patients; mean age was 22.1 years (range 7–46 years). Localizations were cervical (3), cervicothoracic (2), thoracic (3), lumbar (4), and sacrum (6). Tumor was localized on the left side in 11 cases, on the right side in 2, and at midline in 5. The two most common clinical features were axial pain (14 patients) and radicular pain (8 patients). Nine patients had no neurological symptoms, while six patients had motor weakness due to root compression, and three patients had motor weakness due to cord compression.

Mean duration of symptoms was 9 months (3 months–3 years). Preoperative findings of patients were summarized in Table 1.

**Table 1:** Pre-operative findings of patients

No.	Age, sex	Localization	Side	WBB stage	Symptom duration (m)	Symptoms and findings
1	17, F	C2	Left	3–7 ABCD	3	Neck pain, no neurology
2	15, F	C6	Left	1–7 ABCD	6	Neck pain, left arm paresis
3	46, F	C6–C7	Left	1–8, 12 ABCD	4	Neck pain, left arm weakness
4	7, M	C7–T2	Left	1–12 ABCD	3	Back pain, paraplegia
5	8, M	T1–T2	Right	1, 2, 5–12 ABCD	9	Neck pain, radicular pain, no neurology
6	10, M	T7–T8	Midline	1–3, 10–12 ABCD	1	Paraplegia
7	40, M	T11	Left	8–11 ABCD	1	Back pain, no neurology
8	18, M	T12–L1	Left	1–3, 12 ABCD	4	Paraparesis
9	30, M	L2	Midline	4–10 ABCD	24	Low back and leg pain, no neurology
10	18, M	L3	Left	3–7 ABCD	3	Low back and left leg pain, monoparesis
11	17, M	L4	Left	1–4, 12 ABCD	5	Left leg pain, no neurology
12	17, F	L5	Midline	1, 2, 11, 12 ABC	36	Low back pain, no neurology
13	15, M	L5-sacrum	Right	1–12 ABCD	3	Low back and left leg pain, paraparesis
14	13, F	L5-sacrum	Left	2–6 ABCD	12	Low back and left leg pain, no neurology
15	22, F	L5-sacrum	Midline	2–11, ABCD	12	Low back pain, paraparesis
16	29, M	Sacrum	Left	2–8 ABCD	24	Low back pain, no neurology
17	32, F	Sacrum	Left	1–8, 11, 12 ABCD	12	Left leg pain, left monoparesis
18	43, M	Sacrum	Midline	1–12 ABCD	1	Low back and leg pain, no neurology

*M* Male, *F* Female

**Fig. 1:**
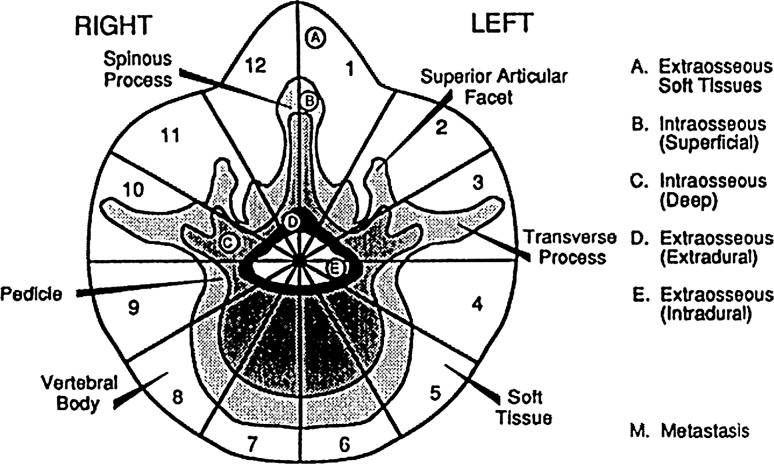
WBB (Weinstein, Boriani, Biagnini) Surgical Staging System. The transverse extension of the vertebral tumor is described with reference to 12 radiating zones (numbered 1–12 in a clockwise order) and to five concentric layers (A–E, from the paravertebral extraosseous compartments to the dural involvement). The longitudinal extent of the tumor is recorded according to the levels involved. From Boriani [9]

Direct radiology disclosed bone erosion in 15 cases. MRI was carried out in 17 patients; one patient underwent CT myelography for diagnosis. Among 17 patients diagnosed with MRI, bone edema was present in six cases. Vascularization was moderate in nine patients, prominent in six, and there were no signs of vascularization in two cases. Upon radiological examination with CT scan or MRI, canal compression was verified in 13 patients: 5 were mild, three were moderate, and 6 were severe. There was no canal compression in four patients. Paravertebral soft tissue mass was determined in 15 cases. There were fluid–fluid levels in 10 cases (Table 2). According to Weinstein, Boriani, Biagini [8, 9] (WBB, Fig. 1) surgical staging, 17 cases were stage ABCD and one patient was stage ABC.

**Table 2:** Radiological findings of patients

No.	Source of radiology	Localization	Plain radiogra.	Canal compression	Fluid–fluid levels	Bone edema in MRI	Para vertebral mass	Vascularization in MRI
1	X-ray, CT, MRI	C2	Normal	+	Yes	No	Yes	++
2	X-ray, CT, MRI	C6	Erosion	–	Yes	Yes	Yes	+++
3	X-ray, CT, MRI	C6–C7	Erosion	+	Yes	Yes	Yes	++
4	X-ray, MRI	C7–T2	Erosion	+++	No	No	Yes	+++
5	X-ray, CT, MRI	T1–T2	Erosion	–	Yes	No	Yes	++
6	X-ray, MRI	T7–T8	Erosion, fracture	+++	No	Yes	Yes	+++
7	X-ray, CT, MRI	T11	Normal	–	No	Yes	No	–
8	X-ray, CT myelography	T12–L1	Erosion	+++	No	NA	Yes	NA
9	X-ray, CT, MRI	L2	Erosion	++	Yes	No	No	++
10	X-ray, CT, MRI	L3	Erosion	++	Yes	Yes	Yes	++
11	X-ray, CT, MRI	L4	Erosion	++	Yes	No	Yes	++
12	X-Ray, CT, MRI	L5	Normal	–	No	No	No	–
13	X-ray, CT, MRI	L5-sacrum	Erosion	+++	Yes	No	Yes	+++
14	X-ray, CT, MRI	L5-sacrum	Erosion	+++	Yes	No	Yes	++
15	X-ray, CT, MRI	L5-sacrum	Erosion	+++	Yes	No	Yes	++
16	X-ray, CT, MRI, DSA	Sacrum	Erosion	+	No	No	Yes	+++
17	X-ray, CT, MRI, DSA	Sacrum	Erosion	+	No	Yes	Yes	+++
18	X-ray, CT, MRI	Sacrum	Erosion	+	No	No	Yes	++

+ Mild, ++ Moderate, +++ Severe, *NA* non available, *DSA* digital subtraction angiography

**Fig. 2:**
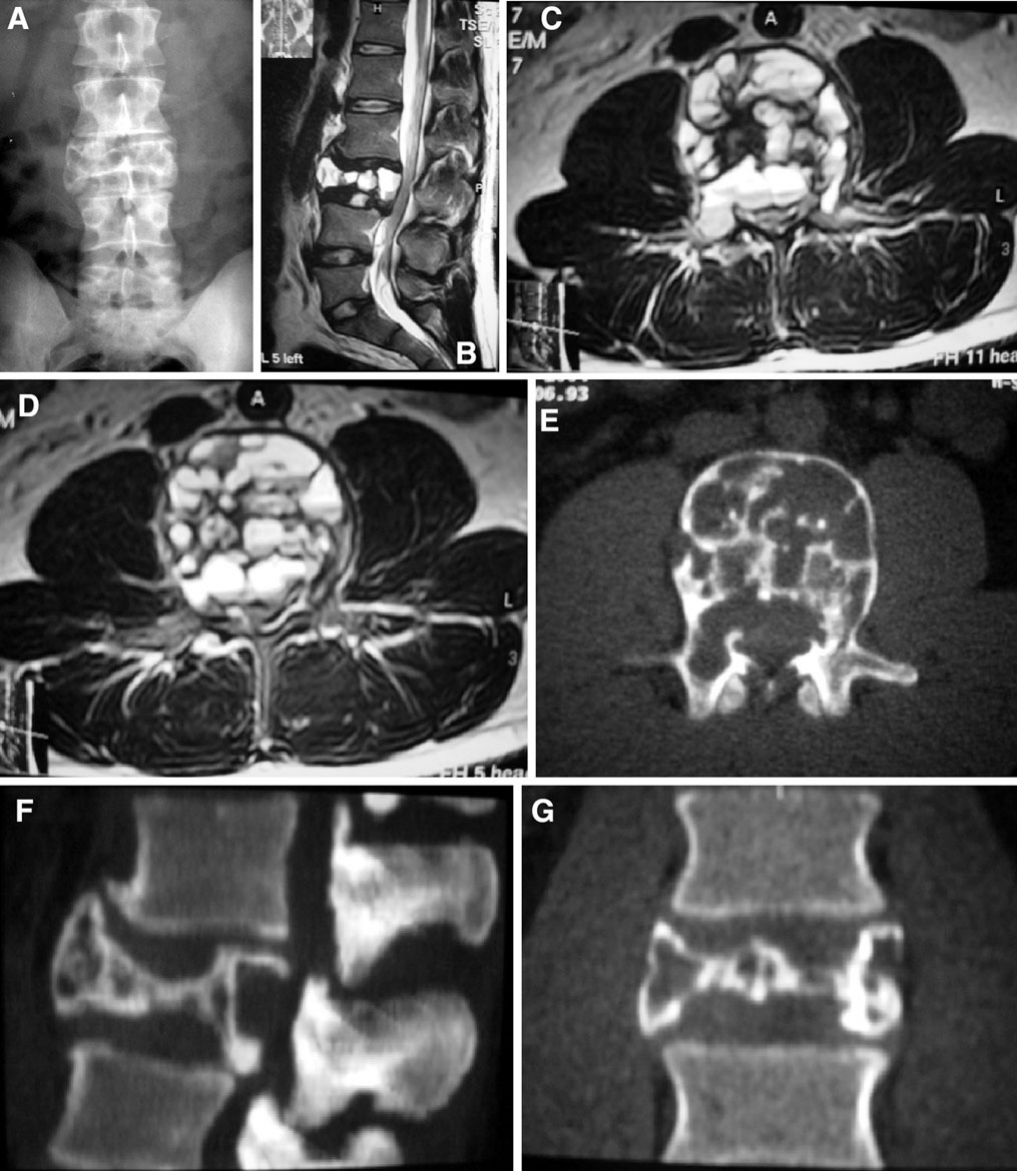

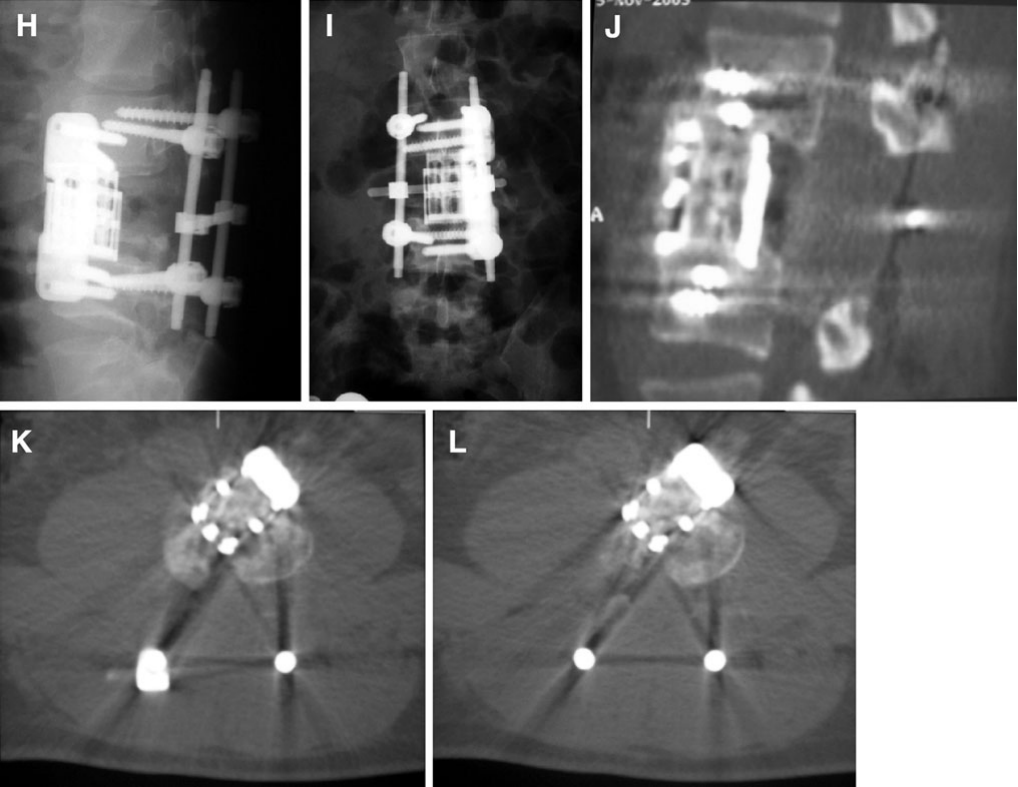
**a–l** Case # 7. A 30-year-old male was admitted to our department with low-back and leg pain for 2 years. There were no neurological deficits. MR and CT images revealed an L3 aneurysmal bone cyst with moderate canal compromise. There were fluid–fluid levels, but no soft tissue mass. WBB scale was 4–10 ABCD. A combined surgical approach (first anterior, then posterior) with gross total removal was performed. A vertebral body cage and posterior pedicle fixation system were used to reconstruct and stabilize the spine. There was no recurrence during the 26-month follow-up time

**Fig. 3:**
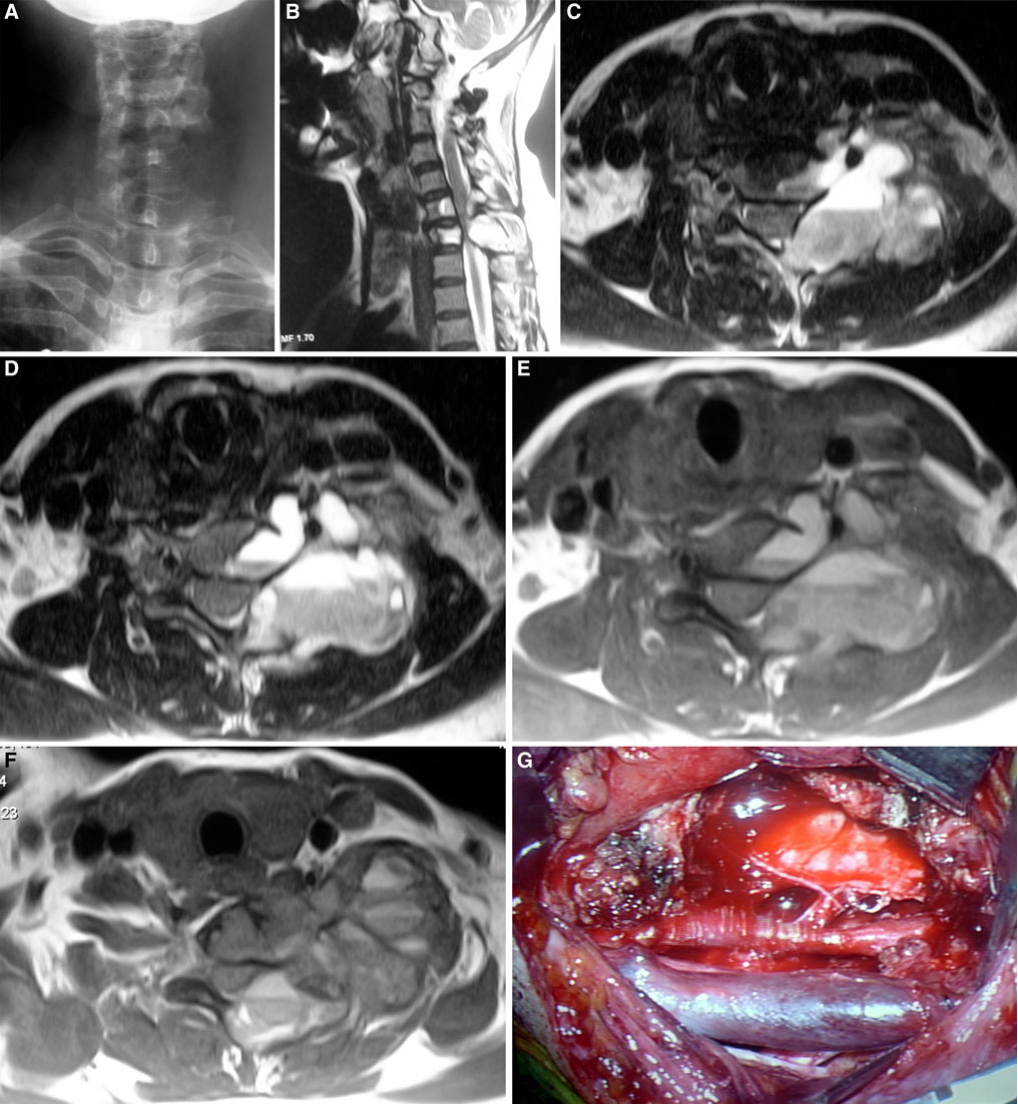

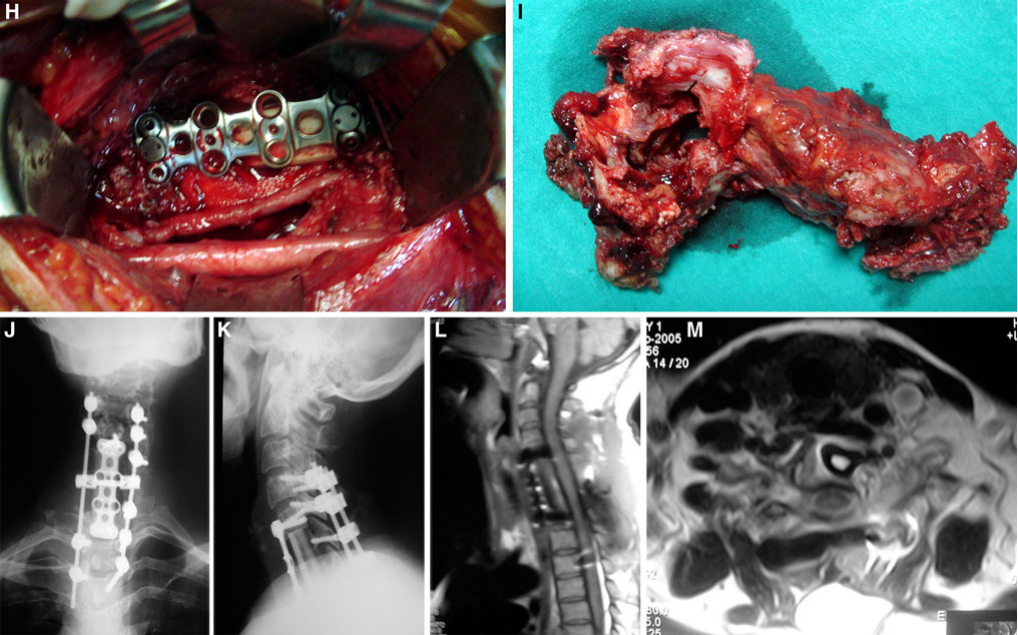
**a**–**m** Case # 2 A 46-year-old female came with neck pain, left arm weakness for 4 months. Neurological examination showed a monoparesis of left upper extremity. MR and CT scans showed a tumor on the left side of C6 and C7 vertebral bodies. C6, C7 body, C7 lamina, pedicle, facet joint, left C6–C7 neural foramina. WBB scale was 1–8, 12 ABCD. A total spondylectomy was performed using a combined posterior, anterior and posterior approach. Spinal reconstruction was achieved using a fibula allograft, anterior cervical plate and posterior lateral mass screw-rod system. There was no recurrence during the 16-month follow-up period

All patients underwent surgery. Total removal could be performed in 13 patients. It was a spondylectomy in one patient. Subtotal resection was performed in five patients. Surgical approaches were posterior alone (11), posterior and lateral (1), and combined anterior-posterior (6) (Figs. 2, 3). Combined approaches were done in one session in five cases, and separate sessions in one case. One patient had a repeat surgery due to recurrence. Six patients were instrumented in addition to tumor removal. Tumor bed was supported with polymethyl methacryate (PMMA) in three patients and with autografts and cage in four patients. On last follow-up, 13 patients have no evidence of disease and five cases are alive with disease (Table 3). As complication, one patient had cerebrospinal fluid (CSF) collection at the site of incision and two patients had significant bleeding during surgery which needed blood transfusion.

**Table 3:** Treatment of patients

No.	Localization	Surgery and approach	No. of surgeries	Tumor removal	Implant, graft	Complication	Follow-up (months)	Last status
1	C2	Post and lat	1	Total	Post fixation, Ant cage and autograft	None	66	NED
2	C6–C7	Comb. post-ant	1	Total	Anterior plate and autograft, posterior screw and rod	None	118	NED
3	C6	Comb. ant-post	1	Subtotal	Posterior plate	CSF collection	90	AWD
4	C7–T2	Post then ant	2	Total	Anterior plate	None	158	NED
5	T1–T2	Comb. post-ant	1	Total	Posterior and anterior plate	None	79	NED
6	T7–T8	Post	1	Total	None	None	202	NED
7	T11	Post	1	Total	None	None	55	NED
8	T12–L1	Post	1	Subtotal	None	None	257	AWD
9	L2	Comb. ant-post	1	Total	Cage, pedicle fixation	None	99	NED
10	L3	Post	1	Total	None	None	136	NED
11	L4	Post	1	Total	None	None	77	NED
12	L5	Post	1	Total	None	None	155	NED
13	L5-sacrum	Post	1	Subtotal	PMMA	None	115	NED
14	L5-Sacrum	Post	1	Total	PMMA	None	57	NED
15	L5-Sacrum	Post	1	Subtotal	PMMA	Over bleeding	45	AWD
16	Sacrum	Post	1	Subtotal	None	None	147	AWD
17	Sacrum	Comb. post-ant	1	Total	None	Over bleeding	83	AWD
18	Sacrum	Post	1	Total	None	None	86	NED

*CSF* cerebrospinal fluid, *NED* no evidence of disease, *AWD* alive with disease, *PMMA* Polymethyl methacryate

## Discussion

The prevalence of ABCs is 1.4 cases per 100,000 individuals, and they constitute approximately 1 % of all bone tumors [2, 7]. The lesions primarily occur in the first two decades of life, with slight women predominance [10]. In this study, mean age was 22.1 years similar with the literature but we have a male predominance (61 %).

ABCs are benign, highly vascular, locally aggressive tumors and recurrence rates after curettage were reported equal or less than 50 % [1, 3]. Spontaneous regression of the tumor is uncommon [11]. Malghem [12] has reported spontaneous healing in three patients.

ABCs have a predilection for the lumbar spine in the series of Boriani and De Kleuver [7, 13]. In contrast, in Papagelopoulos’ and Vergel de Dios’ series, cervical and thoracic spine were involved more than lumbar spine [3, 5]. In our series, sacrum and lumbar spine were involved more than others.

The combination of radiographs, CT scans, and MRI is diagnostic in many cases. Characteristic ballooning of the posterior elements with a thin rim may be shown on plain radiographs [14]. CT imaging reveals multiloculated lytic lesions with multiple internal septations, pathologic fracture or vertebral body collapse. CT scans are also useful for planning of possible instrumentation landmarks during surgery [15]. On MR imaging, ABCs usually demonstrate a thin, well defined rim of low signal intensity in the periphery and they are seen as multiseptate lesions. Usually each lobule represents different signal characteristics giving the tumor a heterogenous appearance. Both CT and MRI are important diagnostic tools for planning the surgical management [7]. We performed both CT and MR for the diagnosis.

Fluid–fluid levels can be seen in the ABC, but this finding is not specific for ABCs. This appearance is also seen in the other bone lesions, which contain areas of hemorrhage or necrosis such as telangiectatic osteosarcoma, giant cell tumor, and chondroblastoma [16]. Differential diagnosis of ABCs includes giant cell tumor, chondroblastoma, chondromyxoid fibroma, fibrosarcoma, telangiectatic osteosarcoma, fibrous dysplasia, simple bone cyst, osteoblastoma, and plasmocytoma [4, 17]. Keenan et al. [18] reported that in their series of patients the incidence of fluid–fluid levels was 85 %. However, in our study fluid–fluid levels were present only in 10 of 18 cases (55 %). This may be because we only evaluated spinal ABCs whereas Keenan et al. have included ABCs originating from the whole skeleton. The question whether spinal ABCs show less frequent fluid–fluid levels than the other parts of the skeleton should be answered through further imaging studies. Another point was to determine whether the nature of ABCs (having fluid–fluid levels versus solid) have influenced the results of the surgical treatment. However, there was no significant difference of the recurrence rates of cystic or solid type of aneurysmal bone cysts.

Although CT an MR are diagnostic methods for many cases, it is noted that in the literature, biopsy is necessary for confirmation, since many bone lesions can have a similar appearance [19]. However, it must be performed cautiously for sometimes needle biopsies can cause complications because the material obtained may consist of mostly blood elements. To prevent such complications, open biopsy and frozen sections were recommended to establish the diagnosis [20]. In this study, biopsy was performed in six cases and we did not see any complication.

Histological examination is definitely necessary to confirm the differential diagnosis. The histology of ABC is typically characterized by cavernous channels surrounded by a spindle cell stroma with osteoclast like giant cells and osteoid production [21]. There are some hypotheses in the literature that the tumor is the result of either hemorrhage into the tumor, or a vascular disturbance of the bone, or improper repair of a traumatic subperiosteal hemorrhages [22].

Treatment of ABC is also controversial. The options for treatment are curettage with or without bone grafting, complete excision, arterial embolization, intralesional drug injections (steroid and calcitonin), and radiation [6, 8]. Early diagnosis and appropriate surgical treatment of ABCs in the spine remain the key factors to successful management [23]. Total excision with or without instrumentation is the optimal approach for local control of tumor and it prevents recurrence [19]. We performed 13 total and 5 subtotal excision with 5 recurrences in this series.

PMMA injection (vertebroplasty or kyphoplasty) may be used to reinforce the bony defects after curettage [24]. Three patients in this study were also reinforced by PMMA injections after removal. There are also reports that the injections of calcitonin or methyl prednisolone inside the ABC cavities are safe procedures with no side effects [14]. Radiotherapy was recommended in inoperable cases, but it has numerous and severe complications including osteonecrosis, gonodal damage, myelopathy and induction of osteosarcoma [3, 6]. Also, preoperative embolization may be performed to minimize intraoperative blood loss [19, 25]. In 2010, Rossi et al. [26] reported their experiences and they considered selective arterial embolization is a less invasive, more feasible, effective and repeatable alternative method to standard surgical treatments. However, we did not use that method in any case in this series.

Recurrence is reported in 10–44 % of the cases, and usually rare when the tumor is excised completely [3]. Ninty percent of recurrences occur within 2 years. Thus, post-treatment follow-up should be at least 24 months [1, 3, 7]. In this series, 13 of 18 patients had a radical surgical removal. We detected four recurrences in subtotal excision group (4/5), and one recurrence in total excision group (1/13). Hay et al. [4] reported that there were no recurrences when total excision was performed, and a 25 % recurrence rate after partial excision. Total excision of large tumors results in bony instability, and instrumentation is necessary to maintain structural integrity [27]. It is also reported that careful preoperative planning is important for management of post excision spinal instability [19]. In this series, six patients have undergone spinal stabilization to prevent spinal deformity and instability.

## Conclusions

Early diagnosis and appropriate surgical treatment of aneurysmal bone cysts in the spine remain the key factors to successful management. Although an effective spinal decompression and stabilization can be achieved by partial or subtotal excisions, recurrence rate is significantly lower in case of total excision. Complete tumor removal would provide a cure for this agressive pathology in long term follow-ups.
